# NLRP3 inflammasome as a therapeutic target in doxorubicin-induced cardiotoxicity: role of phytochemicals

**DOI:** 10.3389/fphar.2025.1567312

**Published:** 2025-04-17

**Authors:** Xiao-Peng Zhao, Lian Duan, Qian-Ru Zhao, Xing Lv, Nai-Yuan Tian, Sheng-Lei Yang, Kun Dong

**Affiliations:** ^1^ College of Exercise and Health, Shenyang Sport University, Shenyang, China; ^2^ China Volleyball College, Beijing Sport University, Beijing, China; ^3^ College of Physical Education, Yanshan University, Qinhuangdao, China; ^4^ Shenyang Sports Research and Medical Center, Shenyang Sports Development Center, Shenyang, China; ^5^ Department of Rehabilitation, Yantai Affiliated Hospital of Binzhou Medical University, Yantai, China; ^6^ School of Sports Medicine and Rehabilitation, Beijing Sport University, Beijing, China

**Keywords:** phytochemicals, NLRP3 inflammasome, pyroptosis, DOX-induced cardiotoxicity, myocardial injury

## Abstract

Doxorubicin (DOX) has received widespread attention as a broad-spectrum antitumor drug. However, it has been a recognized challenge that long-term DOX injections can lead to severe cardiotoxicity. There are numerous interventions to DOX-induced cardiotoxicity, and the most cost-effective is phytochemicals. It has been reported that phytochemicals have complex and diverse biological properties, facilitating the mitigation of DOX-induced cardiotoxicity. DOX-induced cardiotoxicity has numerous pathological mechanisms, and the nod-like receptor family pyrin domain-containing 3 (NLRP3) inflammasome-mediated cardiomyocyte pyroptosis is one of them. This review initially presents an overview of the pathological mechanisms that underlie cardiotoxicity induced by DOX. Subsequently, we present a comprehensive elucidation of the structure and activation of the NLRP3 inflammasome. Finally, we provide a detailed summary of phytochemicals that can mitigate DOX-induced cardiotoxicity by influencing the expression of the NLRP3 inflammasome in cardiomyocytes.

## 1 Introduction

Doxorubicin (DOX) is recognized for its remarkable antitumor efficacy (Agudelo et al., 2014; Xu et al., 2024). However, it is essential to acknowledge that extended administration of DOX can result in significant organ damage, particularly affecting the heart (Carvalho et al., 2009; Wu et al., 2022; Zhao et al., 2023). Available clinical data suggests that prolonged administration of DOX leads to reduced left ventricular ejection fraction (LVEF), arrhythmias and in severe cases, even heart failure (Lipshultz et al., 2002; Volkova and Russell, 2011; Milano et al., 2014; Gupta et al., 2019; Tan et al., 2021; Yun et al., 2021; Harding et al., 2023; Hu et al., 2023; Zhu M. et al., 2024). Recent epidemiologic data indicates that the toxicity of DOX on the heart increases with dose, with a probability of heart failure exceeding 40% for patients when the cumulative dose reaches 700 mg/m^2^ (Gianni et al., 2008; Curigliano et al., 2016). This highly morbid and lethal disease imposes a significant strain on the family of patient and society. Therefore, it is urgent to find appropriate ways to intervene in DOX-induced cardiotoxicity.

Contemporary treatments for DOX-induced cardiotoxicity mainly involve clinical drugs (Zilinyi et al., 2018). Although their efficacy is unquestionably assured, most patients cannot afford the high cost, while long-term administration can lead to adverse effects. Currently, there are several studies indicating that phytochemicals can enhance cardiovascular health (Ditano-Vázquez et al., 2019; Wen et al., 2024). Moreover, numerous investigations have confirmed the potential efficacy of this cost-effective medication in mitigating DOX-induced cardiotoxicity (Abushouk et al., 2017).

DOX-induced cardiotoxicity encompasses various pathological processes, with the nod-like receptor family pyrin domain-containing 3 (NLRP3) inflammasome-mediated cellular pyroptosis being among them and currently one of the most extensively researched (Rawat et al., 2021; Yang et al., 2022). It has been extensively documented that many phytochemicals can reduce DOX-induced cardiotoxicity by affecting the NLRP3 inflammasome and thereby ameliorating cardiomyocyte pyroptosis. This review will summarize and discuss this aspect in detail.

When screening the core literature, we used the following search terms: (((heart*) OR (cardia*) OR (myocardi*) OR (cardiomyocyte*) OR (cardiomyoblast*) OR (cardiomyopathy) OR (H9c2) OR (cardiotoxicity)) AND ((Doxorubicin) OR (DOX))) AND ((NLRP3) OR (nucleotide-binding oligomerization domain-like receptor protein 3)). Using this search strategy, we screened out all research articles that included phytochemicals.

## 2 The overview of doxorubicin-induced cardiotoxicity: from the perspective of pathological mechanisms

Recent studies have been devoted to digging deeper into the pathological mechanisms of DOX-induced cardiotoxicity. Although there has been significant progress, there are still many controversial contents. In this section, we will condense the pathological mechanisms of DOX-induced cardiotoxicity based on the contents of the existing literature.

The first pathological mechanism we will discuss is oxidative stress (Songbo et al., 2019). According to the available studies, DOX can induce oxidative stress in cardiomyocytes in three manners. At the very beginning, DOX can damage the “energy supply centers” in cardiomyocytes, i.e., mitochondria, which can cause the accumulation of reactive oxygen species (ROS) (Octavia et al., 2012). Second, DOX can induce ROS production by affecting Fe^2+^ homeostasis in cardiomyocytes (Sangweni et al., 2022). Finally, DOX can also promote ROS accumulation in cardiomyocytes by increasing nicotinamide adenine nucleotide phosphate (NADPH) oxidase, which in turn induces oxidative stress injury in cardiomyocytes (Priya et al., 2017). Multiple programmed cell deaths are also significant for DOX-induced cardiotoxicity (Christidi and Brunham, 2021). Cardiomyocyte apoptosis is one of them (Qu et al., 2022). Two pathways mainly participate in DOX-induced cardiomyocyte apoptosis. The first is the endogenous pathway (i.e., the mitochondrial pathway), in which DOX can lead to DNA fragmentation by disrupting the outer mitochondrial membrane (OMM) in cardiomyocytes, which can cause the leakage of cytochrome C (Cyt C), which recruits caspase-9 and in turn activates caspase-3 and induces its translocation to the nucleus (Kitazumi and Tsukahara, 2011). The exogenous apoptotic pathway is mainly closely linked to the activation of caspase-8. DOX can activate caspase-3/7 by activating caspase-8, which in turn leads to the development of cardiomyocyte apoptosis (Font-Belmonte et al., 2020). Cardiomyocyte ferroptosis is also one of them (Tai et al., 2023). Available evidence suggests that DOX can induce cardiomyocyte ferroptosis by inducing a massive accumulation of Fe^2+^, thereby disrupting iron homeostasis within cardiomyocytes (Zhao et al., 2023). Meanwhile, excessive Fe^2+^ accumulation induces the Fenton Reaction, which leads to a massive accumulation of lipid peroxides, causing secondary damage (Koppenol, 2001; Kaźmierczak-Barańska et al., 2020; Ai et al., 2021). Cellular autophagy, a particular form of programmed cell death, also participates in DOX-induced cardiotoxicity (Shabalala et al., 2017). In addition, the induction of cellular inflammation is also an important factor in myocardial injury caused by DOX (Vitale et al., 2024). According to relevant reports, DOX can induce the expression of pro-inflammatory factors [e.g., interleukin-1 beta (IL-1β), tumor necrosis factor-alpha (TNF-α), etc.], which induces myocardial cell inflammation (El-Agamy et al., 2019; Li et al., 2019). Of course, many other pathological mechanisms are involved besides the above. For example, DOX can induce endoplasmic reticulum dysfunction in cardiomyocytes, leading to the accumulation of unfolded or misfolded proteins, which in turn induces endoplasmic reticulum stress injury (Sun et al., 2024). DOX also causes damage to mitochondria in cardiomyocytes, leading to mitochondrial dysfunction (Wu et al., 2022). The pathological mechanism of DOX-induced cardiotoxicity may also be related to epigenetic, Ca^2+^ overload and disturbed energy metabolism (Chen et al., 2017; Rawat et al., 2021; Dantas et al., 2022).

**FIGURE 1:**
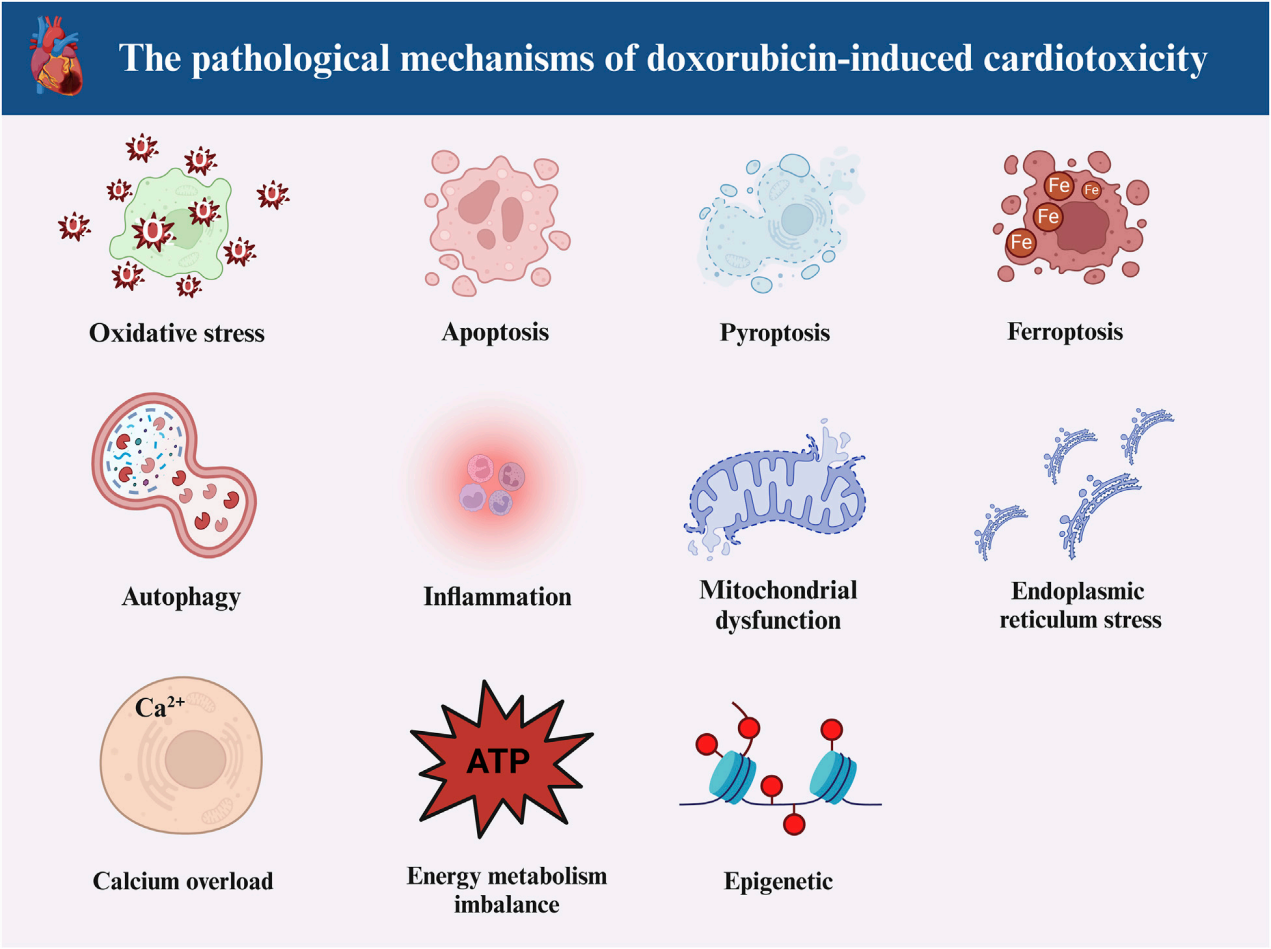
The main pathological mechanisms of doxorubicin-induced cardiotoxicity. Doxorubicin-induced cardiotoxicity involves multiple pathological mechanisms. The main can include oxidative stress, apoptosis, ferroptosis, pyroptosis, autophagy, inflammation, endoplasmic reticulum stress, epigenetics, Ca^2+^ overload, disturbed energy metabolism and mitochondrial dysfunction (Created with BioRender.com).

Notably, cellular pyroptosis mediated by NLRP3 inflammasome has received much attention, and many reports suggest that DOX-induced cardiotoxicity is intimately linked to the activation of NLRP3 inflammasome-mediated cardiomyocytes pyroptosis. Therefore, our review will target NLRP3 inflammasome-mediated cardiomyocyte pyroptosis and provide insights into phytochemicals to ameliorate DOX-induced cardiotoxicity. Figure 1 illustrates the pathological processes of DOX-induced cardiotoxicity.

## 3 The structure and activation of NLRP3 inflammasome

Nucleotide-binding structural domains Leucine-rich repeat proteins (NLRs) constitute a family of pattern recognition receptors (PRRs) predominantly engaged in innate immune defense mechanisms, responding to various damage-associated molecular patterns (DAMPs) and pathogen-associated molecular patterns (PAMPs) (Singh and Jha, 2018; Xia et al., 2023). Notably, there are four subgroups within the NLR family: NLRA, NLRB, NLRC, and NLRP (Xia et al., 2023). Activated NLRPs can form inflammasomes, of which the NLRP3 inflammasome is representative (Wicherska-Pawłowska et al., 2021; Xia et al., 2023). Available reports suggest that the NLRP3 inflammasome comprises three parts: NLRP3 (sensor), apoptosis-associated speckle-like protein (ASC) (adaptor) and caspase-1 (effector) (Li S. et al., 2021). Nucleotide-binding and oligomerization domain (NACHT), C-terminal leucine-rich repeats (LRRs) and an N-terminal pyrin domain (PYD) are the three primary components of NLRP3 (Platnich and Muruve, 2019; Yi, 2020; Li S. et al., 2021; Xia et al., 2023). ASC contains two domains: one at the N-terminal end, the PYD, and the other, the C-terminal caspase recruit domain (CARD) (Platnich and Muruve, 2019; Yi, 2020). Pro-caspase-1 possesses a three-part structure comprising a CARD at the N-terminal, p10 at the C-terminal and p20 centrally located (Platnich and Muruve, 2019; Yi, 2020).

**FIGURE 2:**
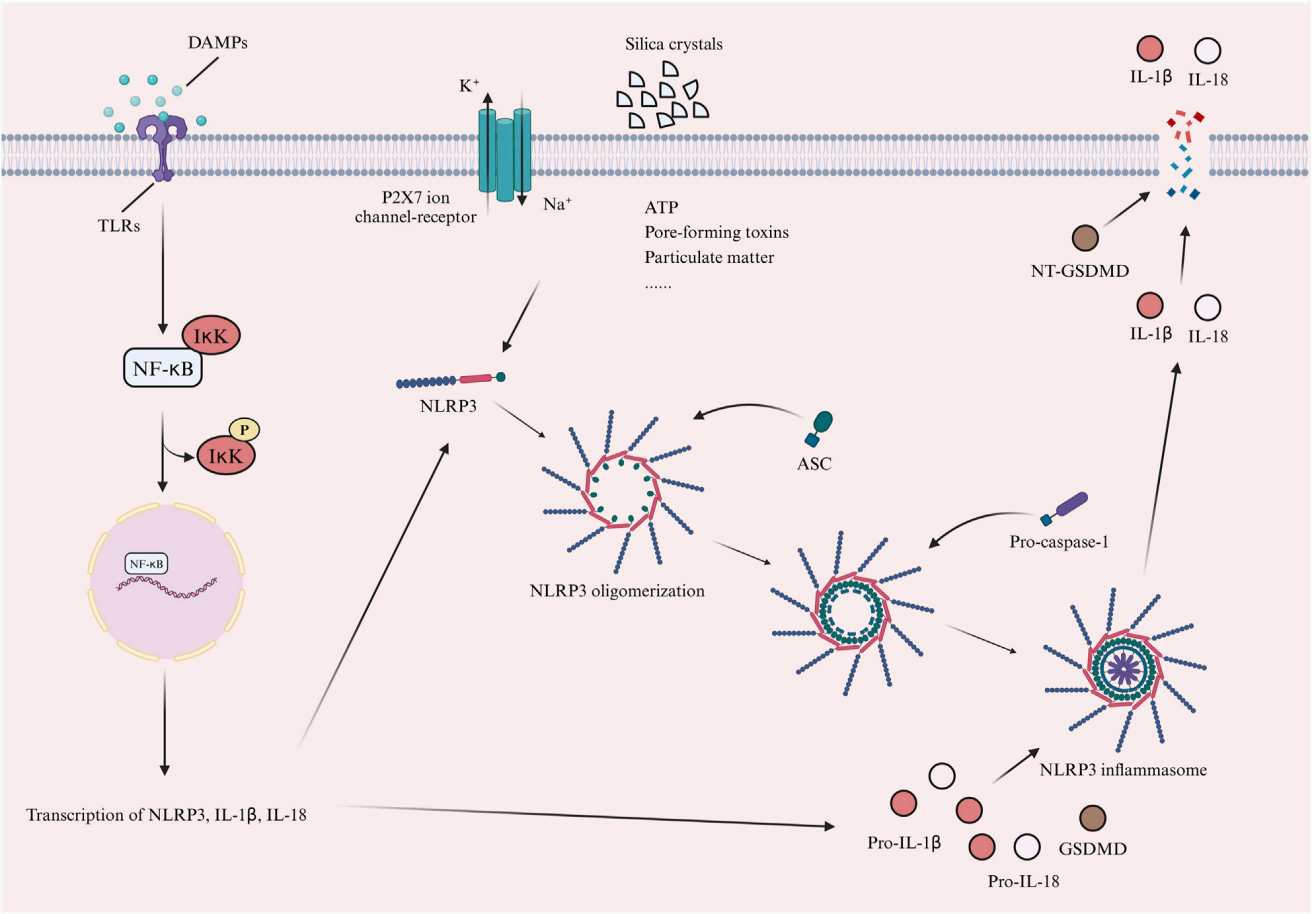
The activation process of NLRP3 inflammasome. The activation of NLRP3 inflammasome depends on two main signals. First is the “priming” signal. This signal is primarily mediated by a variety of PRRs, including TLRs. These receptors recognize PAMPs or DAMPs, thereby triggering the nuclear translocation of NF-κB. This process subsequently induces the transcription of NLRP3, IL-1β, and IL-18. The next is the “triggering” signal. The signal promotes NLRP3 oligomerization and NLRP3 interacts with ASC. ASC binds to pro-caspase-1 to form the NLRP3 inflammasome. Pro-caspase-1 is cleaved into active caspase-1, which activates pro-IL-1β, pro-IL-18 and GSDMD. Activated NT-GSDMD forms membrane pores, releasing IL-1β and IL-18 and inducing pyroptosis. This signal is triggered by factors like ion flow (K^+^ efflux, Na^+^ influx), ATP, pore-forming toxins, particulate matter and silica crystals (Created with BioRender.com). (PRRs, pattern recognition receptors; PAMPs, pathogen-associated molecular patterns; DAMPs, damage-associated molecular patterns; TLRs, toll-like receptors; NF-κB, nuclear factor kappa B; IκK, IkappaB kinase; IL-1β, interleukin-1beta; NLRP3, nod-like receptor family pyrin domain-containing 3; ASC, apoptosis-associated speckle-like protein; GSDMD, gasdermin-D; ATP, adenosine triphosphate; P2X7, purinergic 2X7).

The NLRP3 inflammasome rests by forming a double-loop structure (Andreeva et al., 2021). The NLRP3 inflammasome cannot be activated by the basal level of NLRP3 (Sharma and Kanneganti, 2021). Based on the available studies, it is found that the activation of NLRP3 inflammasome mainly relies on two different signals (Sharma and Kanneganti, 2021; Toldo et al., 2022; Xia et al., 2023). The first is the “priming” signal (Xia et al., 2023). This signal is mainly involved by a variety of PRRs (toll-like receptors (TLRs) as well as multiple cytokine receptors, including the TNF receptor and the IL-1 receptor), which recognize PAMPs and DAMPs, thereby triggering the nuclear translocation of nuclear factor kappa B (NF-κB), which in turn induces the transcription of NLRP3, IL-1β and IL-18 (Sharma and Kanneganti, 2021; Toldo et al., 2022; Xia et al., 2023). This is immediately followed by the “triggering” signal (Sharma and Kanneganti, 2021; Toldo et al., 2022; Xia et al., 2023). This signal allows for the oligomerization of NLRP3 through the NACHT, and the PYD in NLRP3 is primarily responsible for connecting with the PYD in the ASC. This interaction, in turn, makes it easier for the ASC to bind to pro-caspase-1, ultimately forming the NLRP3 inflammasome (Ludlow et al., 2005; Tattoli et al., 2007; Kumar et al., 2009; Rathinam et al., 2012; Latz et al., 2013; Lu et al., 2014; Motta et al., 2015; Toldo et al., 2015; Yin et al., 2015; Murphy et al., 2016; Yang et al., 2016; Pavillard et al., 2017; Arterbery et al., 2018; Komada et al., 2018; Li et al., 2018; Toldo and Abbate, 2018; Hausmann et al., 2020; Mezzaroma et al., 2021). At the same time, pro-caspase-1 undergoes cleavage, resulting in the release of active caspase-1. This released caspase-1 then activates pro-IL-1β, pro-IL-18 and gasdermin-D (GSDMD) (Toldo et al., 2022). The formation of membrane pores by activated N-terminal GSDMD (NT-GSDMD) leads to the release of IL-1β and IL-18 from the intracellular space, which ultimately leads to the induction of cellular pyroptosis (Shao et al., 2007; Liu et al., 2016; Toldo and Abbate, 2018; Abbate et al., 2020). “Triggering” signal is induced by many factors, such as ion flow (K^+^ efflux, Na^+^ influx), adenosine triphosphate (ATP), pore-forming toxins, particulate matter, silica crystals, etc. (Sharma and Kanneganti, 2021). Figure 2 illustrates the activation pathway of the NLRP3 inflammasome.

## 4 Phytochemicals ameliorate doxorubicin-induced cardiotoxicity by inhibiting NLRP3 inflammasome-mediated cardiomyocyte pyroptosis

There are numerous reports on phytochemicals ameliorating DOX-induced cardiotoxicity. He et al. has shown that phytochemical could ameliorate DOX-induced cardiotoxicity by modulating cardiomyocyte autophagy (He et al., 2021). Lin et al. demonstrate that phytochemical could ameliorate DOX-induced cardiotoxicity by affecting cardiomyocyte oxidative stress (Lin and Wang, 2023). NLRP3 inflammasome-mediated pyroptosis of cardiomyocytes is a significant pathological mechanism of DOX-induced cardiotoxicity. Therefore, in this section, based on the available literature, we will classify and summarize which phytochemicals can ameliorate DOX-induced cardiotoxicity by inhibiting NLRP3 inflammasome-mediated cardiomyocyte pyroptosis. Table 1 provides a detailed summary of this section.

**TABLE 1:** Phytochemicals ameliorate doxorubicin-induced cardiotoxicity by affecting NLRP3 inflammasome-mediated cardiomyocyte pyroptosis.

Phytochemical						
Category	Name	PubChem CID	Molecular formula	Structure	Intervention does	Intervention route
Polyphenol	Curcumin	969516	C_21_H_20_O_6_	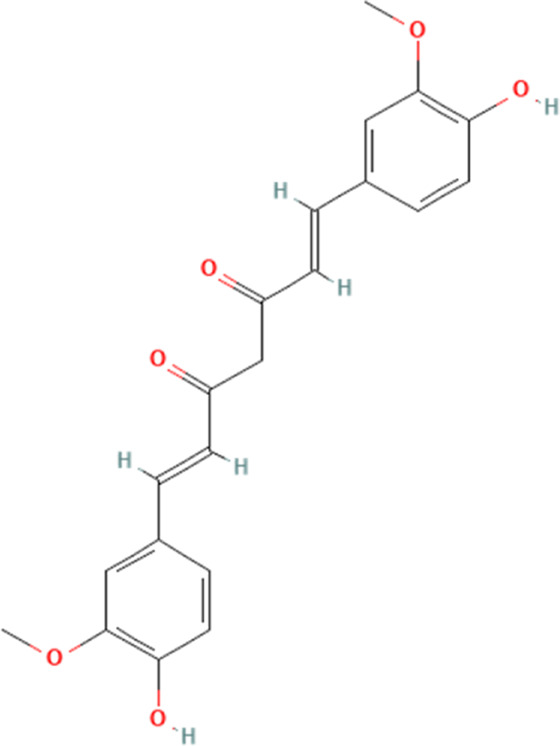	100, 200, 400 mg/kg for 16 days	IG
					10 μM for 24 h	N/A
	Resveratrol	445154	C_14_H_12_O_3_	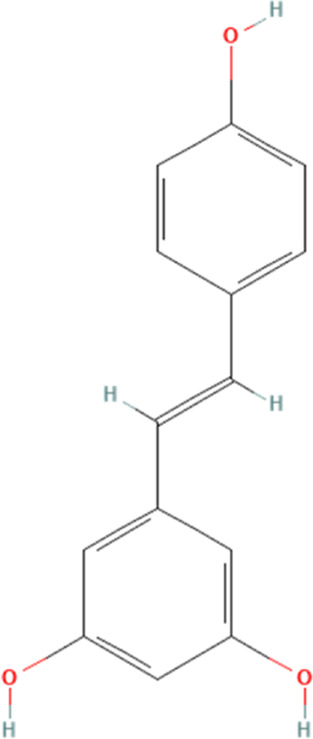	320 mg/kg/day for 5 weeks	PO
	Honokiol	72303	C_18_H_18_O_2_	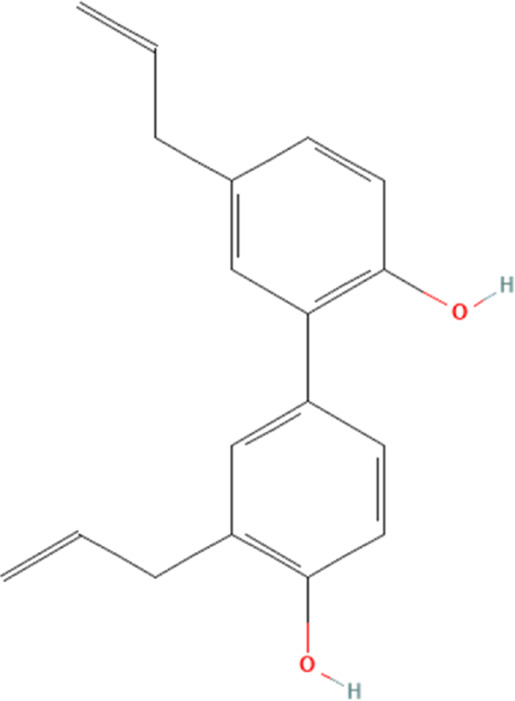	2.5, 5 μM for 24 h	N/A
	Amentoflavone	5281600	C_30_H_18_O_10_	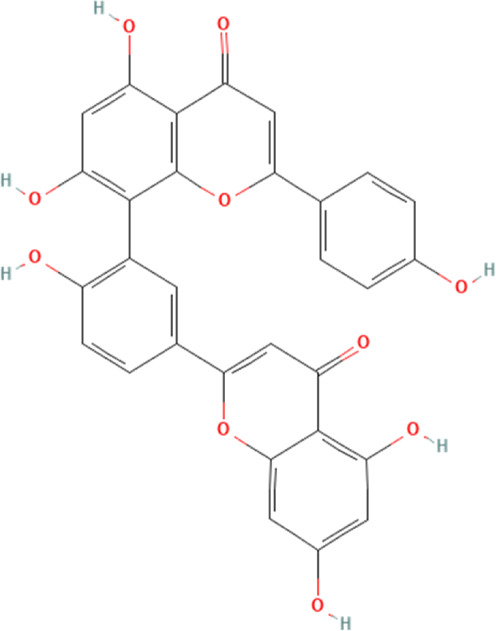	20 mg/kg	N/A
					20 μM for 24 h	N/A
Flavonoid	Myricetin	5281672	C_15_H_10_O_8_	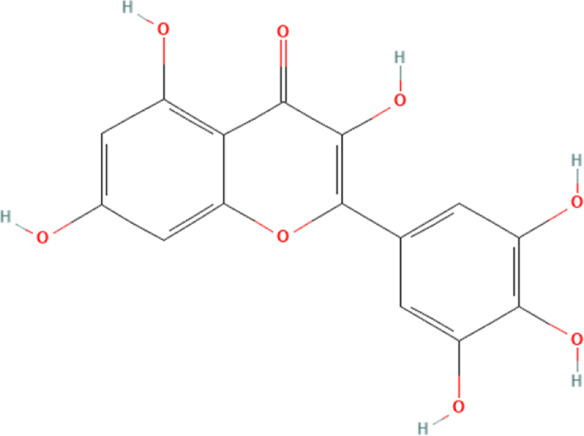	5, 25, 50 mg/kg/day for 1 week	PO
	Pinocembrin	68071	C_15_H_12_O_4_	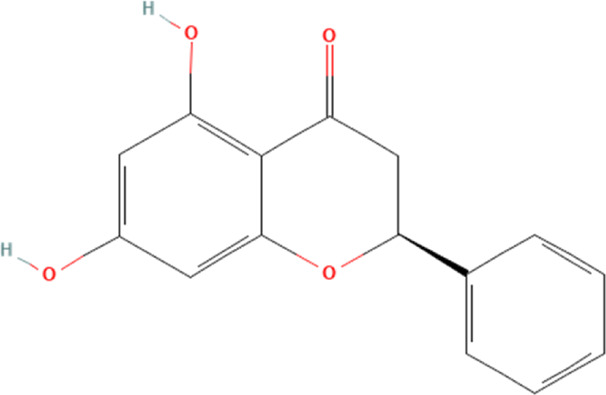	5 mg/kg every other day for 4 weeks	IP
					1 μM for 48 h	N/A
	Dihydromyricetin	161557	C_15_H_12_O_8_	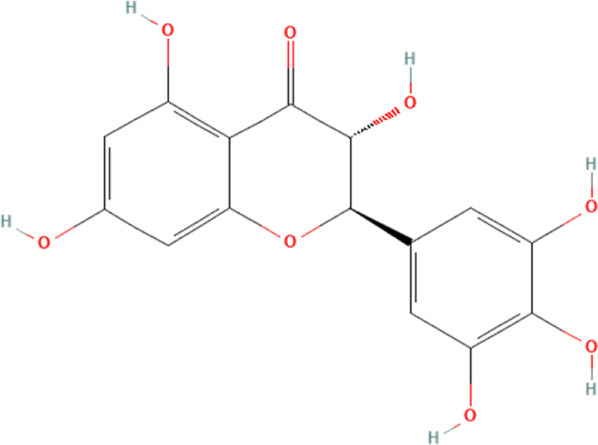	100, 200 mg/kg/day for 6 weeks	PO
					50 μM for 24 h	N/A
	Hyperoside	5281643	C_21_H_20_O_12_	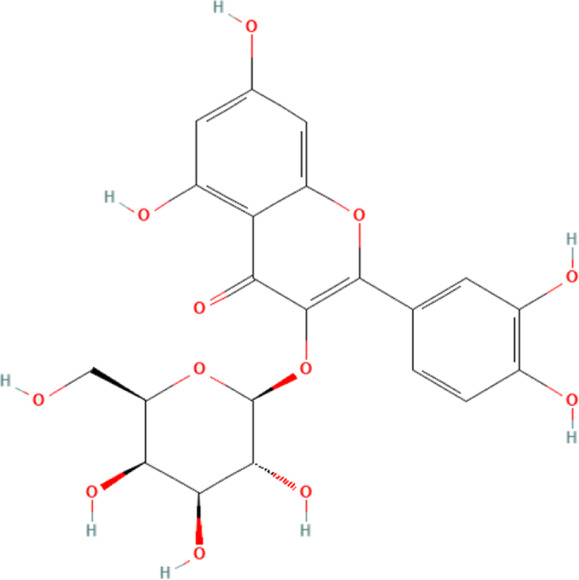	15, 30 mg/kg/day for 7 days	PO
					100, 200 μM for 48 h	N/A
	Calycosin	5280448	C_16_H_12_O_5_	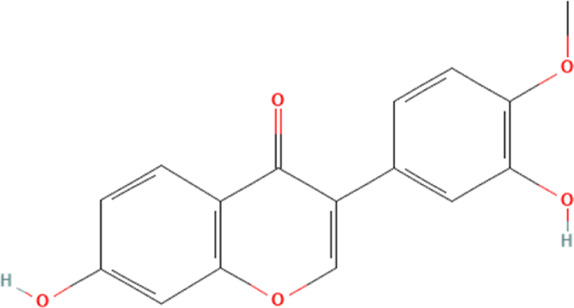	50 mg/kg every other day for 4 weeks	IP
					20 μg/mL for 1 h	N/A
	Calycosin	5280448	C_16_H_12_O_5_	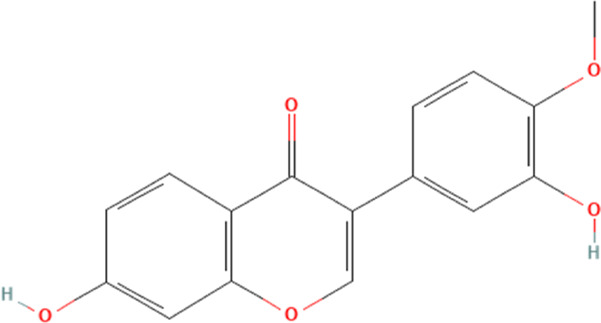	50, 100 mg/kg for 7 days	IP
					50, 100, 200 μM for 24 h	N/A
	Cynaroside	5280637	C_21_H_20_O_11_	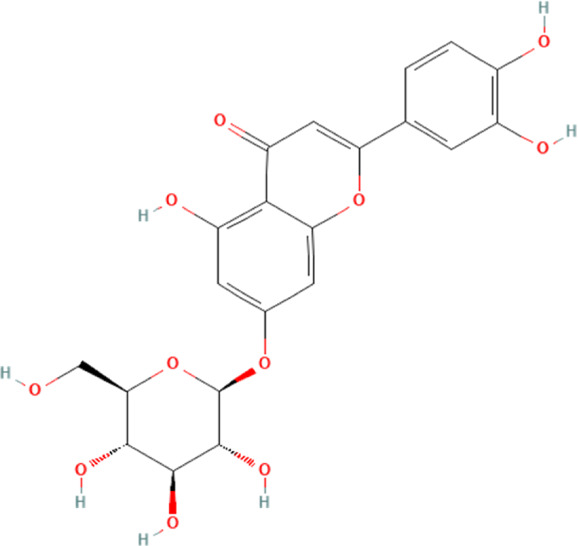	10, 50 mg/kg/day for 9 days	IP
Terpenoid	Carnosic acid	65126	C_20_H_28_O_4_	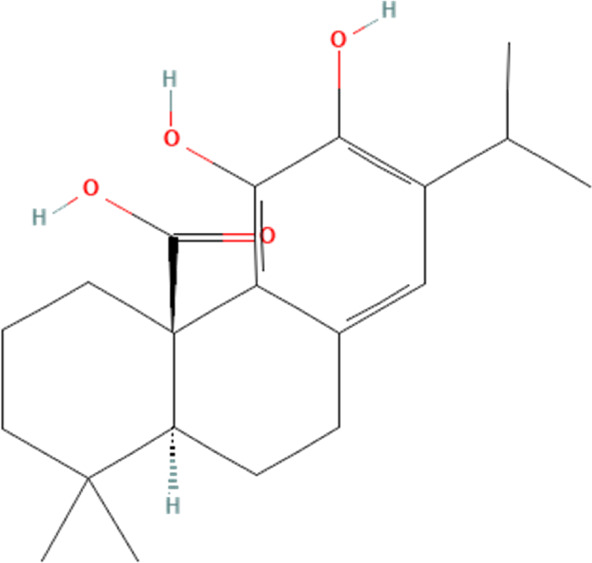	40 mg/kg/day for 4 weeks	PO
					20 μM for 24 h	N/A
	α-Bisabolol	252403102	N/A	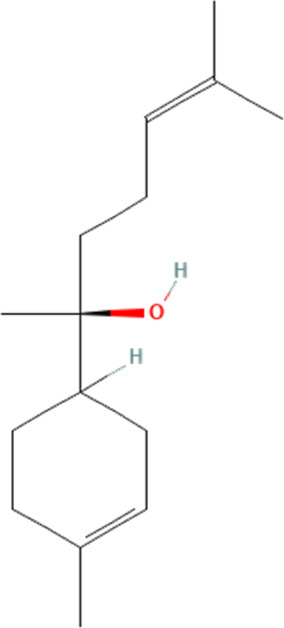	25 mg/kg twice 1 day for 5 days	PO
	Andrographolide	5318517	C_20_H_30_O_5_	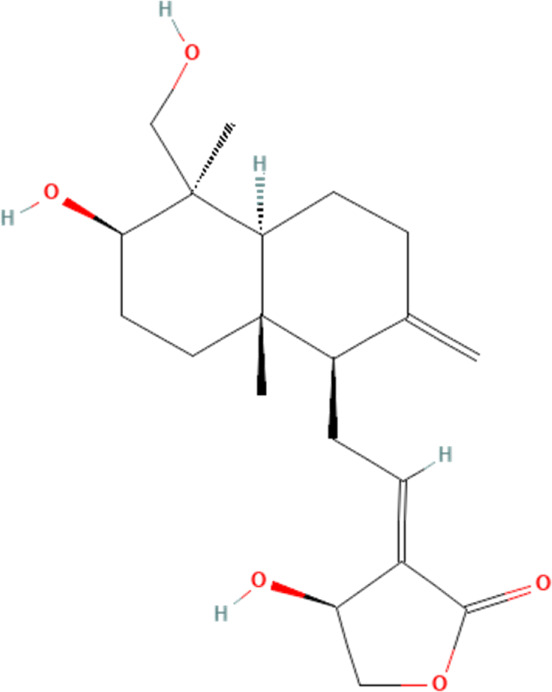	25, 50 mg/kg/day for 17 days	IG
					5, 10 μM for 24 h	N/A
	Nerolidol	5284507	C_15_H_26_O	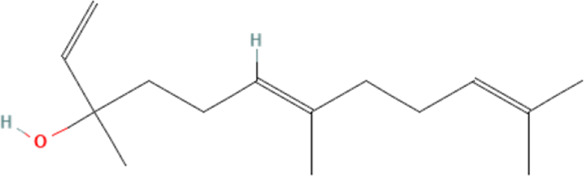	50 mg/kg/day for 5 weeks	PO
Polysaccharide	Polyguluronic acid	481148167	N/A	N/A	12.5, 25, 50 mg/kg for 2 weeks	IP
					12.5, 25, 50 μg/mL for 24 h	N/A
	Fuzi polysaccharide	N/A	N/A	N/A	50, 100, 200 mg/kg for 14 days	IG
Saponin	Astragaloside IV	13943297	C_41_H_68_O_14_	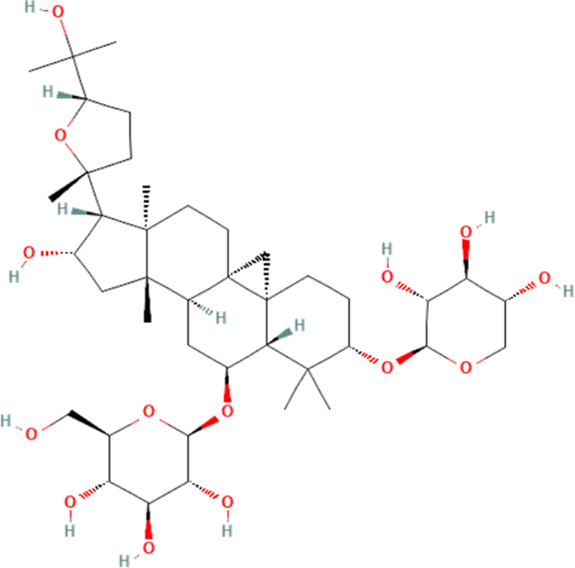	40 mg/kg for 4 weeks	IG
	Astragaloside IV	13943297	C_41_H_68_O_14_	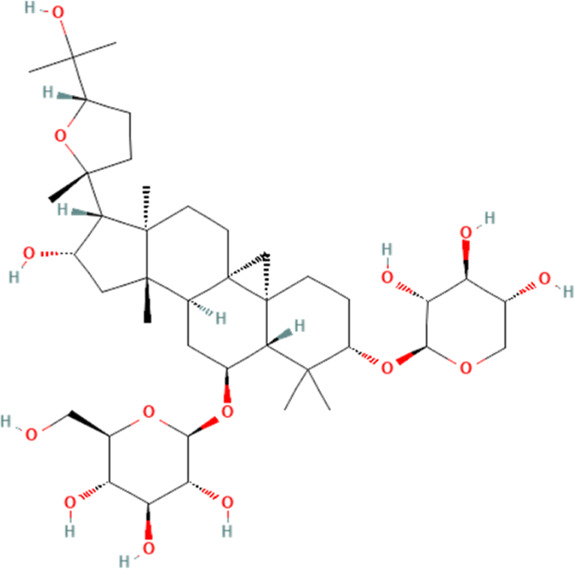	20 mg/kg/day for 5 days/week for 6 weeks	IG
					1 μM for 72 h	N/A
Other small molecule compounds	Fraxetin	5273569	C_10_H_8_O_5_	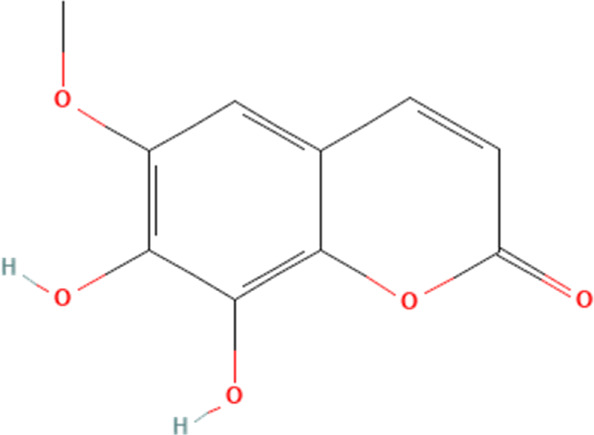	40, 80 mg/kg/day for 1 week	IG
	Betaine	247	C_5_H_11_NO_2_	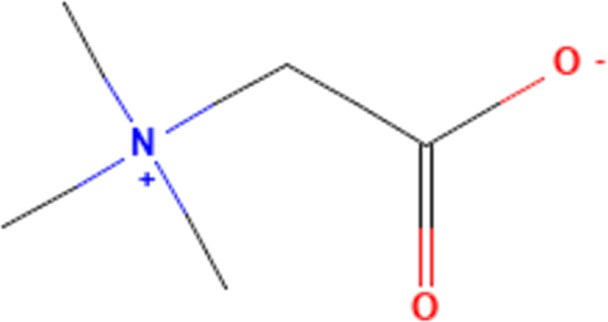	50, 100, 200 mg/kg once a day for 2 weeks	PO

Model				Signaling pathway	Intervention outcome	References
*In Vivo*/*In Vitro*	Animal/Cell	Intervention does	Intervention route			
*In vivo*	Kunming mice	3 mg/kg every other day with a cumulative dosage of 24 mg/kg	IP	NLRP3 inflammasome-mediated cardiomyocyte pyroptosis	HW/BW ↑; CK, LDH, AST ↓; Pumping function of the heart ↑; Cardiac contractility ↑	Yu et al. (2020)
*In vitro*	H9c2	2 μM for 24 h	N/A			
*In vivo*	C57BL/6J mice	4 mg/kg for 3 weeks	IP	NLRP3 inflammasome-mediated cardiomyocyte pyroptosis	Blood pressure ↓; The area of perivascular fibrosis ↓; Myocardial tissue damage ↓	Maayah et al. (2021)
*In vitro*	H9c2	0.1 μM for 48 h	N/A	TXNIP/NLRP3 inflammasome-mediated cardiomyocyte pyroptosis	Cardiomyocyte mortality ↓	Huang et al. (2020)
*In vivo*	C57BL/6J mice	3 mg/kg every other day for 2 weeks	IP	STING/NLRP3 inflammasome-mediated cardiomyocyte pyroptosis	Cardiomyocyte mortality ↓; Pumping function of the heart ↑; Hemodynamics ↑; CK-MB, LDH, BNP ↓; Myocardial tissue damage ↓; The area of myocardial fibrosis ↓; Myocardial pathologic hypertrophy ↓	Fang et al. (2023)
*In vitro*	Primary ventricular cardiomyocytes	1 μM for 24 h	N/A			
*In vivo*	C57BL/6J mice	15 mg/kg for 1 time	IP	NLRP3 inflammasome-mediated cardiomyocyte pyroptosis	cTnI, AST, BNP, LDH ↓; TC, TG ↓; Myocardial tissue damage ↓; The area of myocardial fibrosis ↓	Li et al. (2024)
*In vivo*	C57BL/6J mice	20 mg/kg for 1 time	IV	Nrf2/SIRT3/NLRP3 inflammasome-mediated cardiomyocyte pyroptosis	Pumping function of the heart ↑; Myocardial tissue damage ↓; The area of myocardial fibrosis ↓; CK, LDH ↓; Cardiomyocyte mortality ↓	Gu et al. (2021)
*In vitro*	H9c2	1 μM for 48 h	N/A			
*In vivo*	SD rats	2.5 mg/kg/week for 6 weeks	IV	SIRT1/NLRP3 inflammasome-mediated cardiomyocyte pyroptosis	Cardiomyocyte mortality ↓; The area of myocardial fibrosis ↓; Myocardial pathologic hypertrophy ↓; Pumping function of the heart ↑	Sun et al. (2020)
*In vitro*	H9c2	5 μM for 24 h	N/A			
*In vivo*	C57BL/6J mice	3 mg/kg every other day with a cumulative dosage of 12 mg/kg	IP	NLRP3 inflammasome-mediated cardiomyocyte pyroptosis	Cardiomyocyte mortality ↓; Myocardial pathologic hypertrophy ↓; cTnT, CK, CK-MB, LDH ↓; Pumping function of the heart ↑	Wei et al. (2023)
*In vitro*	Rat primary cardiomyocytes	1 μM for 48 h	N/A			
*In vivo*	C57BL/6J mice	5 mg/kg for 4 weeks	IP	NLRP3 inflammasome-mediated cardiomyocyte pyroptosis	Pumping function of the heart ↑; The area of myocardial fibrosis ↓; Myocardial tissue damage ↓; Cardiomyocyte mortality ↓	Zhang et al. (2022)
*In vitro*	H9c2	1 μM for 24 h	N/A			
*In vivo*	Kunming mice	15 mg/kg for 1 time	IP	SIRT1/NLRP3 inflammasome-mediated cardiomyocyte pyroptosis	Cardiomyocyte mortality ↓; AST, LDH ↓; Myocardial tissue damage ↓	Zhai et al. (2020)
*In vitro*	H9c2	5 μM for 24 h	N/A			
*In vivo*	C57BL/6J mice	5 mg/kg/week for 3 weeks	IP	NLRP3 inflammasome-mediated cardiomyocyte pyroptosis	ALP, AST, LDH, CK-MB ↓; Myocardial tissue damage ↓; Myocardial pathologic hypertrophy ↓; ANP, BNP, Myh7, cTnT, cTnI, Actc1 ↓	Zou et al. (2024)
*In vivo*	C57BL/6J mice	5 mg/kg/week for 3 weeks	IP	Nrf2/NLRP3 inflammasome-mediated cardiomyocyte pyroptosis	Pumping function of the heart ↑; HW/TL ↓; The area of myocardial fibrosis ↓; Myocardial tissue damage ↓; CK-MB, cTnT, LDH ↓; Cardiomyocyte mortality ↓	Hu et al. (2023)
*In vitro*	Neonatal rat ventricular myocytes	1 μM for 24 h	N/A			
*In vivo*	Wistar rats	12.5 mg/kg	IP	NLRP3 inflammasome-mediated cardiomyocyte pyroptosis	Body weight ↑; CK, LDH ↓; Hemodynamics ↑	Nagoor Meeran et al. (2023)
*In vivo*	C57BL/6J mice	3 mg/kg every other day for 10 days	IP	NLRP3 inflammasome-mediated cardiomyocyte pyroptosis	Cardiomyocyte mortality ↓; Pumping function of the heart ↑; CK, LDH, CK-MB, cTnT ↓; Myocardial tissue damage ↓	Liu et al. (2024)
*In vitro*	H9c2	1 μM for 24 h	N/A			
*In vivo*	Wistar rats	2.5 mg/kg for 5 weeks	IP	NLRP3 inflammasome-mediated cardiomyocyte pyroptosis	CK, LDH, Troponin-T, Troponin-I ↓; Body weight ↑; HR, SAP, DAP, MAP ↑; HW/BW ↑; The area of myocardial fibrosis ↓	Meeran et al. (2021)
*In vivo*	C57BL/6J mice	10 mg/kg for 1 time	IP	Peli1/NLRP3 inflammasome-mediated cardiomyocyte pyroptosis	Cardiomyocyte mortality ↓; LDH ↓; Myocardial tissue damage ↓	Zhang et al. (2023)
*In vitro*	HL-1	1 μM for 24 h	N/A			
*In vivo*	Kunming mice	2.5 mg/kg for 12 days	IP	NLRP3 inflammasome-mediated cardiomyocyte pyroptosis/IL-6/STAT3-mediated cardiomyocyte apoptosis	Pumping function of the heart ↑; CK-MB, LDH, NT-pro BNP ↓; Myocardial tissue damage ↓	Xiong et al. (2024)
*In vivo*	C57BL/6J mice	Every other day with a cumulative dosage of 28 mg/kg	IP	Nrf2/NLRP3 inflammasome-mediated cardiomyocyte pyroptosis	Pumping function of the heart ↑; CK-MB, LDH, BNP, cTnI ↓; The area of myocardial fibrosis ↓; Myocardial pathologic hypertrophy ↓; HW/BW ↑	Chen et al. (2023)
*In vivo*	C57BL/6J mice	4 mg/kg/week for 4 weeks	IP	SIRT1/AMPK or AKT/NLRP3 inflammasome-mediated cardiomyocyte pyroptosis	Pumping function of the heart ↑; CK-MB, LDH ↓; Myocardial tissue damage ↓	Tian et al. (2024)
*In vitro*	H9c2	1 μM for 24 h	N/A			
*In vivo*	Wistar rats	4 mg/kg for twice/week for 2 weeks	IP	NLRP3 inflammasome-mediated cardiomyocyte pyroptosis	Pumping function of the heart ↑; The area of myocardial fibrosis ↓; Myocardial tissue damage ↓; CK-MB, LDH, Troponin I, NT-pro BNP ↓	Kabel et al. (2021)
*In vivo*	NMRI mice	2.5 mg/kg for 15 mg/kg	IP	NLRP3 inflammasome-mediated cardiomyocyte pyroptosis	CK-MB, LDH, Troponin I ↓; Myocardial tissue damage ↓	Mohammadpour et al. (2024)

IG, intragastrical administration; IP, intraperitoneal injection; N/A, not applicable; NLRP3, the Nod-like receptor family pyrin domain-containing 3; HW, heart weight; BW, body weight; CK, creatine kinase; LDH, lactate dehydrogenase; AST, aspartate transaminase; PO, per os; TXNIP, thioredoxin-interacting protein; STING, stimulator of interferon genes; CK-MB, creatine kinase-myoglobin; BNP, brain natriuretic peptide; cTnI, cardiac troponin I; TC, cholesterol; TG, triglycerides; IV, intravenous injection; Nrf2, nuclear factor erythroid-2-related factor 2; SIRT3, sirtuin 3; cTnT, cardiac troponin T; ALP, alkaline phosphatase; ANP, atrial natriuretic peptide; Myh7, myosin heavy chain 7; Actc1, actin alpha cardiac muscle 1; TL, tibia length; HR, heart ratio; SAP, systolic arterial pressure; DAP, diastolic arterial pressure; MAP, mean arterial pressure; Peli1, pellino 1; STAT3, signal transducer and activator of transcription 3; IL-6, interleukin 6; AMPK, AMP-activated protein kinase; AKT, protein kinase B.

### 4.1 Polyphenol

Polyphenols are organic compounds distinguished by many phenolic units and are extensively found in diverse plants (Salucci et al., 2017; Tian et al., 2021). There are numerous reports that polyphenols can be involved in relieving DOX-induced cardiotoxicity by modulating multiple biological functions (Gu et al., 2015; Yi et al., 2024). Based on the available literature, four polyphenolic phytochemicals could ameliorate DOX-induced cardiotoxicity by inhibiting NLRP3 inflammasome-mediated cardiomyocyte pyroptosis.

#### 4.1.1 Curcumin

Curcumin is a bright yellow polyphenolic compound isolated and extracted from turmeric, which has significant anti-inflammatory and anti-oxidative stress properties (Vollono et al., 2019; Ms et al., 2020; D'Andurain et al., 2023). Available studies suggest its crucial function in mitigating DOX-induced cardiotoxicity. According to a report, curcumin could reduce inflammation and oxidative stress in cardiomyocytes, thereby ameliorating DOX-induced cardiotoxicity (Ibrahim Fouad and Ahmed, 2022). Interestingly, an existing report suggests that curcumin’s amelioration of DOX-induced cardiotoxicity is also strongly associated with its inhibition of NLRP3 inflammasome (Yu et al., 2020). This study has shown that DOX has a number of negative effects on the heart, including a decrease in heart weight, an increase in blood levels of the biomarkers of myocardial injury like creatine kinase (CK), lactate dehydrogenase (LDH), aspartate transaminase (AST) and cardiac troponin I (cTnI) and a noticeable impact on the efficiency and contractility of the cardiac pumping function. Further experiments show that DOX can dramatically raise the production of NLRP3 inflammasome and proteins associated with cell pyroptosis, including caspase-1, IL-1β and IL-18, in the myocardium. This indicates that the cardiac injury induced by DOX is likely associated with the triggering of the NLRP3 inflammasome and its subsequent mediation of cellular pyroptosis in the myocardium. After curcumin intervention, the severe cardiac damage caused by DOX is significantly reversed, and the expression of NLRP3 inflammasome and its downstream-mediated cellular pyroptosis-associated proteins are significantly reduced. This suggests that curcumin’s amelioration of DOX-induced cardiotoxicity may connect to its inhibition of NLRP3 inflammasome and cardiomyocyte pyroptosis. How does curcumin affect DOX-induced expression of NLRP3 inflammasome in cardiomyocytes? Unfortunately, this article does not explore this. However, the effects of curcumin on DOX-induced expression of ROS as well as the phosphatidylinositol 3-kinase (PI3K)/protein kinase B (AKT)/mammalian target of the rapamycin (mTOR) signaling pathway in cardiomyocytes are also examined in this study. Is there a link between curcumin’s altered expression of NLRP3 inflammasome and ROS or PI3K/AKT/mTOR signaling pathway? This will hopefully be confirmed in future studies. Meanwhile, it is noteworthy that there are no clinical reports on the amelioration of DOX-induced cardiotoxicity by curcumin, which may be attributed to its lower oral utilization (Maiti et al., 2007; Chen et al., 2015).

#### 4.1.2 Resveratrol

Resveratrol, a polyphenolic compound, is present in various plants, including grapes and peanuts, is known for its substantial anti-oxidant and anti-aging capabilities and is essential in the treatment of numerous cardiovascular illnesses (Petrovski et al., 2011; Zhang et al., 2021). A recent report demonstrates that resveratrol significantly reduces ferroptosis in cardiomyocytes, thereby ameliorating DOX-induced cardiotoxicity (Chen L. et al., 2024). Interestingly, available evidence suggests that resveratrol can also reduce the severity of cardiac damage caused by DOX by suppressing the NLRP3 inflammasome and cardiomyocyte pyroptosis (Maayah et al., 2021). The study shows that DOX significantly increases blood pressure in mice, induces perivascular fibrosis in the myocardium and severely affects the normal structure of the myocardium. This may be due to the activation of NLRP3 inflammasome and the proteins related to cellular pyroptosis, IL-1β and IL-18. The intervention of resveratrol could well reverse the above pathological effects and significantly reduce the DOX-induced activation of NLRP3 inflammasome and downstream cellular pyroptosis-related proteins. This suggests that resveratrol can significantly ameliorate DOX-induced cardiotoxicity, and this effect may be attributed to the inhibition of NLRP3 inflammasome and downstream cellular pyroptosis-related proteins in cardiomyocytes. However, is there some link between NLRP3 inflammasome and ferroptosis regulated by resveratrol in DOX-induced cardiotoxicity? We propose a novel idea, that whether resveratrol can influence ferroptosis by influencing the expression of NLRP3 inflammasome in DOX-induced cardiomyocytes? This question is still unanswered. However, it has been shown that a significant upstream-downstream relationship between NLRP3 inflammasome and ferroptosis has also been reported (Hacioglu, 2024; Yuan et al., 2024; Zhang et al., 2024). Therefore, there is a theoretical basis for speculating that resveratrol can also affect DOX-induced ferroptosis in cardiomyocytes by influencing the expression of NLRP3 inflammasome, and thus ameliorating DOX-induced cardiotoxicity. Meanwhile, there are no clinical studies on resveratrol ameliorating DOX-induced cardiotoxicity, and resveratrol’s solubility may explain why. Resveratrol has been reported to have a low solubility of 0.005 mg/mL in water, and its chemical structure is unstable when exposed to the intestine and stomach, which leads to its inefficient utilization after oral administration (Francioso et al., 2014; Robinson et al., 2015).

#### 4.1.3 Honokiol

Honokiol, a polyphenolic small molecule compound stemming from the bark of Magnolia officinalis, exhibits many effects, including anti-allergic and anti-anxiety, and is crucial in the treatment of cardiovascular disorders (Munroe et al., 2010; Guillermo et al., 2012; Wang et al., 2018; Liu et al., 2023). A recent report shows that honokiol can significantly ameliorate DOX-induced mitochondrial damage, thereby attenuating DOX-induced cardiotoxicity (Pillai et al., 2017). Another study shows honokiol could ameliorate DOX-induced cardiotoxicity by modulating NLRP3 inflammasome-mediated cardiomyocyte cellular pyroptosis (Huang et al., 2020). This study demonstrates that DOX significantly reduces cardiomyocyte viability and promotes senescence-related proteins such as p16^INK4A^ and p21. This suggests that DOX can increase cardiomyocyte mortality by inducing cardiomyocyte senescence. Further experiments demonstrate that DOX significantly induces the expression of thioredoxin-interacting protein (TXNIP) and NLRP3 inflammasome and its downstream-mediated cellular pyroptosis proteins, such as casapse-1 and IL-1β, in cardiomyocytes. This demonstrates that DOX can also lead to cardiomyocyte death by inducing cardiomyocyte pyroptosis. Honokiol’s intervention significantly ameliorates DOX-induced cellular senescence and NLRP3 inflammasome-mediated cellular pyroptosis. Further experiment overexpressing TXNIP shows that the function of honokiol disappears. This demonstrates that the function of honokiol is closely related to TXNIP and that honokiol attenuates DOX-induced cellular senescence and NLRP3 inflammasome-mediated cellular pyroptosis by inhibiting TXNIP. However, it is noteworthy that this study attributes DOX-induced cardiomyocyte senescence to NLRP3 inflammasome-mediated cellular pyroptosis. We think this is inappropriate. Because the study does not modulate NLRP3 inflammasome to detect cardiomyocyte senescence, it is impossible to determine whether NLRP3 inflammasome-mediated cellular pyroptosis is upstream of cellular senescence.

#### 4.1.4 Amentoflavone

Amentoflavone is a polyphenol compound derived from Selaginella tamariscina (Ma et al., 2024). Available studies have demonstrated that amentoflavone has significant anti-inflammatory and anti-apoptotic properties and is essential in ameliorating several cardiovascular diseases (Qin et al., 2018; Li W. W. et al., 2021). A study has demonstrated that amentoflavone ameliorates DOX-induced cardiotoxicity by attenuating NLRP3 inflammasome-mediated cellular pyroptosis (Fang et al., 2023). This study demonstrates that DOX significantly affects the pumping function and hemodynamics of the heart, as well as significantly inducing cardiac hypertrophy and myocardial fibrosis, with damaging effects on the structure of myocardial tissue. The study also shows that DOX significantly increases blood levels of the markers of myocardial damage, creatine kinase-myoglobin (CK-MB) and LDH. The emergence of these pathologies may be closely associated with increased NLRP3 inflammasome and their downstream-mediated cellular pyroptosis proteins such as caspase-1 and GSDMD, IL-1β and IL-18. This experiment further shows that DOX promotes stimulator of interferon genes (STING) expression. Interestingly, amentoflavone intervention significantly reverses various myocardial pathologic injuries caused by DOX and significantly reduces NLRP3 inflammasome-mediated cellular pyroptosis-related proteins and STING. This suggests that the mechanism of amentoflavone in ameliorating DOX-induced cardiotoxicity may be closely related to its inhibition of NLRP3 inflammasome and its downstream-mediated cardiomyocyte pyroptosis. Further experiments overexpressing STING reveal that the effect of amentoflavone disappears and NLRP3 expression is significantly elevated. This suggests that amentoflavone ameliorates DOX-induced cardiotoxicity by inhibiting STING and, thus, NLRP3 inflammasome, inhibiting cardiomyocyte pyroptosis.

### 4.2 Flavonoid

Flavonoids are a category of vital phytochemicals characterized by polyphenolic structures, frequently found in a variety of plants, fruits, and vegetables as secondary metabolites (Martínez-Coria et al., 2023; Yi, 2023). In recent years, reports on flavonoid phytochemicals ameliorating DOX-induced cardiotoxicity have gradually increased. In the following section, we will summarize six different flavonoid phytochemicals ameliorating DOX-induced cardiotoxicity by inhibiting NLRP3 inflammasome-mediated cardiomyocyte pyroptosis.

#### 4.2.1 Myricetin

Myricetin, a flavonoid phytochemical, is frequently present in various natural plants, especially prunes (Song et al., 2021). According to numerous preclinical investigations, myricetin serves multiple biological purposes and is essential in the amelioration of cardiovascular-related diseases (Zhang et al., 2018; Zhu J. et al., 2024). A study has shown that myricetin can ameliorate DOX-induced cardiotoxicity by modulating NLRP3 inflammasome-mediated cardiomyocyte cellular pyroptosis (Li et al., 2024). This study describes in detail that DOX significantly increases the release of myocardial injury markers such as cTnI, AST, brain natriuretic peptide (BNP) and LDH in the blood and increases the levels of cholesterol (TC) and triglycerides (TG) in the blood. Pathological staining experiments prove that DOX could significantly damage the structure of myocardial tissue and induce the development of myocardial fibrosis. Further experiments demonstrate that DOX significantly induces NLRP3 inflammasome-mediated cellular pyroptosis in myocardial tissues (derived from increased NLRP3, ASC and caspase-1). However, the above pathology is significantly reversed, and the expression of NLRP3 inflammasome and their mediated cellular pyroptosis proteins in cardiomyocytes are significantly reduced after the intervention of low, medium and high doses of myricetin. Therefore, this study implies that myricetin’s effect in mitigating DOX-induced cardiotoxicity is likely associated with its inhibition of NLRP3 inflammasome and cardiomyocyte pyroptosis. It is crucial to acknowledge that there are no clinical cases concerning the amelioration of DOX-induced cardiotoxicity by myricetin, and we believe that based on the broad pharmacological effects of myricetin, future studies should gradually validate their association in clinical studies.

#### 4.2.2 Pinocembrin

Pinocembrin is a flavonoid phytochemical found mainly in Euphorbia, Sparattosperma leucanthum and Pinus heartwood (Rasul et al., 2013). Its widespread pharmacological activity in the anti-cardiovascular disease has been well studied (Li C. et al., 2021). In a study of DOX-induced cardiotoxicity, Gu et al. (2021) find that DOX could significantly affect the pumping function of the heart and can lead to the development of fibrosis and myocardial tissue damage. The study also shows that DOX can increase the release of CK-MB and LDH, markers of myocardial damage, in the blood. Further experiments demonstrate that DOX could inhibit the nuclear translocation of nuclear factor erythroid-2-related factor 2 (Nrf2) and activate the downstream sirtuin 3 (SIRT3)/NLPR3 signaling pathway, which in turn can induce the expression of cellular pyroptosis proteins, such as caspase-1 and GSDMD-N, and ultimately lead to myocardial pyroptosis. In contrast, pinocembrin significantly reverses DOX-inhibited Nrf2 nuclear translocation, thereby inhibiting the SIRT3/NLPR3 signaling pathway and its downstream-mediated cellular pyroptosis proteins, and thus ameliorating DOX-induced cardiotoxicity. Nevertheless, it is pertinent to acknowledge that the relationship between SIRT3 and NLRP3 inflammasome is not demonstrated in this study, and we are aware that many studies have demonstrated that SIRT3 is located upstream of NLRP3 inflammasome. However, it is illogical to conclude the above results directly in this study because SIRT3 is not modulated to detect the expression level of NLRP3 inflammasome.

#### 4.2.3 Dihydromyricetin

Dihydromyricetin is a flavonoid phytochemical widely found in Ampelopsis grossedentata (Wang et al., 2023). One recent study suggests it serves a crucial function in attenuating DOX-induced cardiotoxicity (Sun et al., 2020). As this research demonstrates, DOX can significantly induce myocardial damage, myocardial fibrosis and pathological cardiac hypertrophy. In the meanwhile, the pumping function of the heart is also significantly affected. Additional experimental evidence suggests that myocardial injury may be associated with the induction of NLRP3 inflammasome and cellular pyroptosis. The intervention of dihydromyricetin significantly reverses the above pathology and inhibits the induction of NLRP3 inflammasome and cellular pyroptosis. Interestingly, the study further explores how dihydromyricetin can affect the expression of NLRP3 inflammasome in cardiomyocytes. This study finds that DOX can significantly inhibit the expression of SIRT1, while the expression of SIRT1 is significantly elevated after dihydromyricetin intervention, and the effect of dihydromyricetin is found to disappear after silencing SIRT1. This demonstrates that dihydromyricetin can inhibit the induction of NLRP3 inflammasome by activating the expression of SIRT1 in cardiomyocytes, thereby inhibiting cardiomyocyte pyroptosis and ultimately attenuating DOX-induced cardiotoxicity. Notably, it has been found that dihydromyricetin has a low oral utilization and short half-life, which significantly affects its use in the clinic (Wang et al., 2023).

#### 4.2.4 Hyperoside

Hyperoside is a flavonoid phytochemical found in various plants such as Hypericum monogynum (Hypericaceae), Crataegus pinnatifida (Rosaceae) and Polygonum aviculare (Polygonaceae) (Xu et al., 2022). One available evidence has demonstrated its crucial role in interfering with DOX-induced cardiotoxicity (Wei et al., 2023). This study suggests that DOX significantly promotes cardiac hypertrophy in mice and facilitates the release of myocardial injury biomarkers, such as cardiac troponin T (cTnT), CK, CK-MB and LDH, in the blood, as well as affecting the pumping function of the heart. Subsequent investigations indicate that the cause of myocardial injury may be closely related to the activation of NLRP3 inflammasome and its downstream-mediated cellular pyroptosis. Intervention with hyperoside significantly reverses these pathologies and simultaneously reduces the expression of NLRP3 inflammasome and its downstream-mediated cellular pyroptosis in cardiomyocytes. Therefore, this study concludes that the mechanism of hyperoside intervention in DOX-induced cardiotoxicity may be intricately linked to its suppression of NLRP3 inflammasome and its downstream-mediated cellular pyroptosis. It is worth noting that in this study, hyperoside is administered to mice by gavage. Yuan et al. have examined the impact of hyperoside of different modes of administration on rats, and they have found that the bioavailability of hyperoside in rats is significantly low following gavage administration and that intraperitoneal injection (IP) and intravenous administration (IV) are the best ways to promote the absorption and utilization of hyperoside in the body (Yuan et al., 2021).

#### 4.2.5 Calycosin

Calycosin, a flavonoid phytochemical, is derived mainly from the root of Astragalus membranaceus and a typical phytoestrogen (Han et al., 2018; Deng et al., 2021). Current studies have shown that calycosin has multiple biological functions and is essential in ameliorating several cardiovascular diseases (Pan et al., 2020). A recent study shows that calycosin could ameliorate DOX-induced cardiotoxicity through the inhibition of NLRP3 inflammasome-mediated cardiomyocyte pyroptosis (Zhang et al., 2022). This study shows that DOX significantly damages the pumping function of the heart and induces cardiac fibrosis. Further experiments demonstrate that DOX increases NLRP3 inflammasome and their downstream-mediated cardiomyocyte pyroptosis. Intervention with calycosin reverses these pathologies and reduces NLRP3 inflammasome-mediated pyroptosis in cardiomyocytes. Therefore, this study concludes that the amelioration of DOX-induced cardiotoxicity by calycosin may be closely related to the inhibition of NLRP3 inflammasome in cardiomyocytes. Another study further explores the association between calycosin and NLRP3 inflammasome in cardiomyocytes (Zhai et al., 2020). They demonstrate that the SIRT1 inhibitor significantly promotes the expression of NLRP3 inflammasome in cardiomyocytes, whereas calycosin significantly promotes SIRT1 expression in cardiomyocytes. Therefore, the study suggests that calycosin may inhibit the expression of NLRP3 inflammasome in cardiomyocytes by promoting the expression of SIRT1, thereby inhibiting cardiomyocyte pyroptosis and ameliorating DOX-induced cardiotoxicity. Unfortunately, there are fewer pharmacokinetic studies on calycosin, and most of the current studies are on the co-metabolism of calycosin in combination with other drugs, so the metabolic process for calycosin can only be derived by speculating on its composition (Deng et al., 2021).

#### 4.2.6 Cynaroside

Cynaroside is a flavonoid phytochemical distributed in the honeysuckle plant, and it has potential biological properties such as anti-inflammatory, anti-oxidant and anti-pyroptosis (Zou et al., 2024). A study demonstrates that cynaroside could inhibit DOX-induced cardiomyocyte death by affecting the expression of the NLRP3 inflammasome (Zou et al., 2024). This study shows that DOX can significantly induce structural damage in mouse myocardial tissue and promote the release of myocardial damage markers alkaline phosphatase (ALP), AST, LDH and CK-MB in the blood. Further experiments demonstrate that the myocardial damage caused by DOX is partly due to the activation of NLRP3 inflammasome in cardiomyocytes and its downstream-mediated cardiomyocyte pyroptosis. The intervention of cynaroside significantly reverses the above pathologic effects, and this reversal may result from the inhibition of NLRP3 inflammasome and their downstream-mediated pyroptosis in cardiomyocytes.

### 4.3 Terpenoid

Terpenoids are the biggest class of compounds in natural products, predominantly derived from plants (Huang et al., 2012). The biological functions of terpenoids are well documented. Here, we will summarize four different terpenoids that ameliorate DOX-induced cardiomyocyte pyroptosis, thus improving myocardial injury by affecting the expression of NLRP3 inflammasome in cardiomyocytes.

#### 4.3.1 Carnosic acid

Carnosic acid, a phenolic diterpenoid, is found in Lamiaceae plants, including Rosemary (Rosmarinus officinalis L.) (Chen X. et al., 2024). Available evidence suggests its crucial role in intervening in cardiovascular-related diseases (Sahu et al., 2014; Zhang et al., 2019). One study demonstrates that carnosic acid could mitigate DOX-induced cardiomyocyte death by suppressing NLRP3 inflammasome in cardiomyocytes (Hu et al., 2023). The study shows that DOX significantly induces cardiac pumping dysfunction, disrupts normal myocardial structure and induces myocardial fibrosis in mice. It also promotes the release of biomarkers that indicate damage to the myocardium, such as CK-MB, LDH, and cTnT, in the blood. Subsequent research has shown that DOX-induced cardiotoxicity may be intricately linked to the activation of the NLRP3 inflammasome and its facilitation of cardiomyocyte pyroptosis proteins, including caspase-1, IL-1β, and IL-18. This study also shows that DOX significantly inhibits the expression of Nrf2 and its downstream anti-oxidative stress-related proteins, heme oxygenase-1 (HO-1). Interestingly, intervention with carnosic acid significantly reverses the above pathology, in which the suppression of NLRP3 inflammasome and cellular pyroptosis may have an indispensable role. To explore how carnosic acid could suppress NLRP3 inflammasome, the study silences Nrf2 and finds that the therapeutic effect of carnosic acid disappears after silencing Nrf2. This suggests that carnosic acid ameliorates DOX-induced cardiotoxicity by activating Nrf2 expression in cardiomyocytes, thereby inhibiting the expression of NLRP3 inflammasome and their downstream cellular pyroptosis. It is crucial to recognize that although many natural compounds possess significant pharmacological capabilities, they may also elicit severe side effects or hazardous responses under certain circumstances, with carnosic acid serving as a pertinent example. Several evidences have been targeted to explore the toxic effects of carnosic acid and have demonstrated the presence of acute, chronic, hepatic and renal toxicity of carnosic acid (Dickmann et al., 2012; Wang et al., 2012; Liu et al., 2017). Therefore, the cytotoxicity of carnosic acid must be taken into account when using.

#### 4.3.2 α-Bisabolol

As a monocyclic sesquiterpene alcohol, α-Bisabolol is the primary source of Matricaria chamomilla, and available evidence has suggested that it is present in large quantities of medicinal plants’ essential oils (McKay and Blumberg, 2006; Eddin et al., 2022). The biological functions of α-Bisabolol have been validated, and many studies have demonstrated its ameliorative and palliative effects in various cardiovascular diseases (Meeran et al., 2018; Nagoor Meeran et al., 2019). One study has demonstrated that α-Bisabolol can ameliorate DOX-induced cardiomyocyte death by modulating the expression of NLRP3 inflammasome in cardiomyocytes (Nagoor Meeran et al., 2023). This study shows that DOX significantly increases the blood levels of myocardial injury markers such as CK and LDH and significantly affects the cardiac hemodynamics in rats. Further experiments demonstrate that DOX significantly increases the expression of NLRP3 inflammasome and its downstream-mediated cell pyroptosis-associated proteins, such as caspase-1 and IL-18, in cardiomyocytes. This suggests that the cause of the above myocardial injury by DOX may be closely related to the activation of NLRP3 inflammasome in the myocardium. Intervention of α-Bisabolol can significantly reverse the above effects. This suggests that the mechanism of α-Bisabolol in ameliorating DOX-induced cardiotoxicity is partly related to the inhibition of NLRP3 inflammasome-mediated cellular pyroptosis. Like carnosic acid, α-Bisabolol has toxic effects. Toxic reactions such as sedation, ataxia, dyspnea and affective blunting have been demonstrated after oral α-Bisabolol overdose (Api et al., 2020; Eddin et al., 2022). Therefore, the intervention dosage of α-Bisabolol is an issue that must be focused on in future clinical studies.

#### 4.3.3 Andrographolide

Andrographolide, a labdane diterpene, is mostly sourced from Andrographis paniculata, exhibiting a diverse array of pharmacological effects (Burgos et al., 2020). One available evidence suggests that andrographolide can mitigate DOX-induced cardiotoxicity (Liu et al., 2024). The study claims that DOX induces cardiomyocyte death and promotes the expression of markers of myocardial damage, CK-MB and LDH, in the blood of mice, as well as affecting the pumping function of the heart. Further experiments demonstrate that myocardial injury caused by DOX is closely related to the activation of NLRP3 inflammasome in cardiomyocytes. Andrographolide significantly reverses these pathologies, inhibiting NLRP3 inflammasome and their downstream-mediated cellular pyroptosis in cardiomyocytes. This, therefore, suggests that the mechanism of andrographolide in ameliorating DOX-induced cardiotoxicity has a profound connection with the suppression of NLRP3 inflammasome and their downstream-mediated cellular pyroptosis in cardiomyocytes.

#### 4.3.4 Nerolidol

Nerolidol, a sesquiterpene alcohol, is naturally present in the essential oil of a variety of plants (Lapczynski et al., 2008; Ferreira et al., 2012; Chan et al., 2016). One available evidence suggests that nerolidol has an effect that interferes with DOX-induced cardiotoxicity (Meeran et al., 2021). This study demonstrates that DOX could induce the release of myocardial damage markers CK, LDH and Troponin-T/I in the blood while inducing cardiac fibrosis in mice. Further experiments show that these pathologic effects are closely linked to the activation of NLRP3 inflammasome and their mediated cellular pyroptosis. This study demonstrates that nerolidol can effectively reverse the pathology caused by DOX, and this effect may be intimately connected to its capacity to diminish the expression of the NLRP3 inflammasome and cellular pyroptosis in cardiomyocytes. It is encouraging that the toxic effects of nerolidol are low and that none of the doses used are sufficient to cause severe toxic effects (Chan et al., 2016). Therefore, many translational clinical studies should be on the agenda based on the doses used.

### 4.4 Polysaccharide

Polysaccharides are an important class of biomolecules, consisting of more than 10 monosaccharide molecules condensed by dehydration, and are widely distributed in nature (Zhou and Huang, 2021). Polysaccharides have extensive array of biological characteristics, such as anti-oxidant, anti-viral and anti-inflammatory, and available evidence also suggests that they also play a critical role in ameliorating DOX-induced cardiotoxicity (Chen F. and Huang G., 2018; Chen L. and Huang G., 2018; Liu et al., 2018; Wang et al., 2022). Here, we will summarize two different polysaccharides based on the existing studies that affect the NLRP3 inflammasome, thereby ameliorating DOX-induced cardiomyocyte pyroptosis and consequently ameliorating myocardial injury.

#### 4.4.1 Polyguluronic acid

Polyguluronic acid, a polysaccharide from alginate, has several biological effects, such as anti-inflammatory and anti-oxidants (Dun et al., 2015; Bakhtiari et al., 2019). The evidence suggests its crucial role in intervening in DOX-induced cardiotoxicity (Zhang et al., 2023). This study shows that DOX can induce cardiomyocyte death, significantly affect the normal structure of myocardial tissue in mice and promote the release of LDH, a marker of myocardial injury, in the blood. This phenomenon may be closely related to the activation of NLRP3 inflammasome and its downstream-mediated casapse-1 and GSDMD proteins. Polyguluronic acid could reverse the above pathologic effects and significantly reduce the expression of NLRP3 inflammasome and cardiomyocyte pyroptosis. To investigate how polyguluronic acid reverses the expression of NLRP3 inflammasome, the study finds that DOX significantly activates the expression of Pellino1 (Peli1), while polyguluronic acid significantly inhibits its expression. However, the pathology of cardiomyocytes is found to be significantly reduced after Peli1 inhibition. This suggests that polyguluronic acid may ameliorate cardiomyocyte injury by inhibiting the expression of Peli1, which in turn inhibits the expression of NLPR3 inflammasome, which in turn inhibits cardiomyocyte pyroptosis.

#### 4.4.2 Fuzi polysaccharide

Fuzi polysaccharide is a water-soluble polysaccharide compound derived from Fuzi and has a variety of biological effects (Yan et al., 2010). Available studies have shown its significant contribution to the treatment of cardiovascular diseases (Liao et al., 2013). A report of DOX-induced cardiotoxicity has demonstrated that fuzi polysaccharide could ameliorate DOX-induced cardiomyocyte death by modulating the expression of NLRP3 inflammasome in cardiomyocytes (Xiong et al., 2024). This study shows that DOX can significantly reduce cardiac contractile function and induce the expression of myocardial damage biomarkers, including CK-MB and LDH, in the blood of mice. The study also implies that the activation of the NLRP3 inflammasome in cardiomyocytes may be strongly connected with the cause of myocardial injury and intervention with fuzi polysaccharide significantly reverses the above pathologic effects and reduces the expression of NLRP3 inflammasome and their mediated pyroptosis in the myocardium. Interestingly, the study further reveals that DOX can significantly stimulate the expression of IL-6 while suppressing the expression of signal transducer and activator of transcription 3 (STAT3). IL-6/STAT3 signaling pathway-mediated apoptosis has been demonstrated (Zhou et al., 2021). Therefore, this study suggests that the cause of myocardial injury by DOX may be through activation of NLRP3 inflammasome and, thus, activation of cardiomyocyte pyroptosis, which, in turn, activates the IL-6/STAT3 signaling pathway and thus causes apoptosis. Fuzi polysaccharide ameliorates DOX-induced cardiotoxicity by blocking the above pathway. However, we think that this study does not detect the association between cellular pyroptosis and the IL-6/STAT3 signaling pathway, and it is inappropriate to connect the two directly. Subsequent experiments should detect the expression of the IL-6/STAT3 signaling pathway after cellular pyroptosis has been regulated and then make a corresponding judgment.

### 4.5 Saponin

Saponins, amphiphilic compounds, are composed of carbohydrates and either triterpenoid or steroid aglycone moieties, frequently discovered in herbal and conventional medicines (Juang and Liang, 2020). The biological functions of saponins have been well studied, and saponins have been found to have a very important potential in intervening in cardiovascular-related diseases (Liu et al., 2014; Sun et al., 2016; Yao et al., 2022). Astragaloside IV, a saponin extracted from A. membranaceus Bunge, is rich in biological activities (Zhou et al., 2023). Two available evidences suggest that astragaloside IV carries a significant amount of weight in attenuating DOX-induced cardiotoxicity (Chen et al., 2023; Tian et al., 2024). One study shows that DOX can significantly induce pump dysfunction and promote fibrosis of myocardial tissue in mice. It also could induce the release of biomarkers that indicate injury to the myocardium, such as CK-MB, LDH and cTnI in the blood. Further experiments show that DOX can also induce the expression of NLRP3 inflammasome and cardiomyocyte pyroptosis. Intervention with astragaloside IV significantly reverses these pathologies and significantly reduces the expression of NLRP3 inflammasome and their mediated cell pyroptosis-associated proteins in cardiomyocytes. Notably, the study further explores the association between astragaloside IV and NLRP3 inflammasome in cardiomyocytes and finds that DOX can significantly inhibit the expression of Nrf2 and its downstream anti-oxidant-associated protein Ho-1, which is significantly reversed by astragaloside IV intervention. Therefore, this study concludes that astragaloside IV could inhibit ROS production in cardiomyocytes by activating Nrf2, thereby attenuating the expression of NLRP3 inflammasome and preventing cardiomyocyte pyroptosis. However, this study does not experimentally validate the relationship between Nrf2 and NLRP3, which is a shortcoming. Of course, another report on astragaloside IV further explores how astragaloside IV inhibits the expression of NLRP3 inflammasome in cardiomyocytes (Tian et al., 2024). This study shows that DOX can significantly inhibit SIRT1 expression, which is significantly elevated after astragaloside IV intervention. However, the pharmacologic effect of astragaloside IV is found to be lost after SIRT1 is inhibited. This suggests that astragaloside IV works by activating SIRT1, which in turn inhibits NLRP3 inflammasome, thus inhibiting cardiomyocyte pyroptosis. Interestingly, the study also examines the expression of AMP-activated protein kinase (AMPK) and AKT in cardiomyocytes and demonstrates that both AMPK and AKT are located downstream of SIRT1 and that astragaloside IV inhibits cardiomyocyte pyroptosis by activating SIRT1, which in turn activates either AMPK or AKT, which in turn inhibits the expression of the NLRP3 inflammasome in cardiomyocytes, and thereby inhibits cardiomyocyte pyroptosis, which in turn ameliorate DOX-induced cardiotoxicity. However, since neither AMPK nor AKT are modulated in this study to assess their effect on SIRT1 or NLRP3, this conclusion may be premature.

### 4.6 Other small molecule compounds

In addition to polyphenols, flavonoids, terpenoids, polysaccharides and saponins phytochemicals, several small molecule compounds do not fall into the above categories that can also ameliorate DOX-induced cardiotoxicity by affecting the NLRP3 inflammasome in cardiomyocytes. We will elaborate here. First, fraxetin. Fraxetin is a coumarin phytochemical with a variety of biological functions (Robe et al., 2021). In a study of DOX-induced cardiotoxicity, fraxetin is reported to ameliorate DOX-induced cardiomyocyte pyroptosis by affecting the expression of NLRP3 inflammasome in cardiomyocytes (Kabel et al., 2021). The study reports that DOX significantly increases the levels of myocardial injury markers such as CK-MB, LDH, cTnI and NT-pro BNP in the blood in rats, while significantly affecting the pumping function of the heart and inducing myocardial fibrosis. Further experiments demonstrate that DOX significantly activates the expression of NLRP3 inflammasome in cardiomyocytes. The above pathological effects are significantly reversed by high and low doses of fraxetin, which also reduces the expression of NLRP3 inflammasome in cardiomyocytes. This suggests that the impact of fraxetin in mitigating DOX-induced cardiotoxicity may be closely related to its inhibition of NLRP3 inflammasome-mediated cell pyroptosis. Another report is about betaine. There is a report that betaine, as a type of alkaloid, can also ameliorate DOX-induced cardiomyocyte pyroptosis by affecting NLRP3 inflammasome in cardiomyocytes (Mohammadpour et al., 2024). This study shows that DOX significantly induces the release of markers of myocardial injury, such as CK-MB, LDH and Troponin I, in the blood while affecting the structure of myocardial tissue. Further experiments show that these pathological effects are intimately connected to the activation of the NLRP3 inflammasome in cardiomyocytes, which are significantly reversed by the intervention of betaine, which is intricately associated with the suppression of the NLRP3 inflammasome in cardiomyocytes.

## 5 Future and perspective

This review focuses on NLRP3 inflammasome-mediated pyroptosis in myocardial cells and details the amelioration of DOX-induced cardiotoxicity by various phytochemicals. This work comprehensively delineates the several pathological pathways behind DOX-induced cardiotoxicity, including oxidative stress, programmed cell death, inflammation and endoplasmic reticulum stress, as informed by recent studies. Subsequently, we provide a comprehensive overview of the NLRP3 inflammasome. Finally, based on the existing findings, we summarize in detail that a variety of phytochemicals ameliorate DOX-induced cardiotoxicity by affecting NLRP3 inflammasome-mediated cardiomyocyte pyroptosis.

However, combined with existing research, they have several shortcomings. Primarily, the majority of current research on phytochemicals to mitigate DOX-induced cardiotoxicity has been confined to preclinical investigations and has not progressed to clinical trials. However, combined with our research in the field, the necessary clinical translation is worth pursuing. Second, during phytochemical interventions, different doses may play different roles. In many of the studies, only an experimental dose is set up, which will seriously undermine an in-depth exploration of the biological functions of the phytochemical. Third, according to the existing reports, we find that some studies claim that phytochemicals such as curcumin and resveratrol belong to pan-assay interference compounds (PAINS). In other words, these phytochemicals can interfere with various reactions (not based on a specific interaction between compound molecules and proteins) in the experiment to achieve a “positive” result. These phytochemicals can seriously affect the output of the experimental results. Therefore, experiments with these phytochemicals should be repeated several times to prevent “false-positive” results.

Regarding the above, we call for the collaboration of additional pertinent researchers to implement systematic experimental design and analysis, as well as to develop an effective evaluation system, thereby enhancing the utilization of phytochemicals for the benefit of patients afflicted by DOX-induced cardiotoxicity.
